# Generation of a Fast Healthcare Interoperability Resources (FHIR)-based Ontology for Federated Feasibility Queries in the Context of COVID-19: Feasibility Study

**DOI:** 10.2196/35789

**Published:** 2022-04-27

**Authors:** Lorenz Rosenau, Raphael W Majeed, Josef Ingenerf, Alexander Kiel, Björn Kroll, Thomas Köhler, Hans-Ulrich Prokosch, Julian Gruendner

**Affiliations:** 1 IT Center for Clinical Research Lübeck Germany; 2 Institute for Medical Informatics University Clinic Rheinisch-Westfälische Technische Hochschule Aachen Aachen Germany; 3 Leipzig Research Centre for Civilization Diseases University of Leipzig Leipzig Germany; 4 Federated Information Systems German Cancer Research Center Heidelberg Germany; 5 Complex Data Processing in Medical Informatics Medical Faculty Mannheim Mannheim Germany; 6 Chair of Medical Informatics Friedrich-Alexander University Erlangen-Nürnberg Erlangen Germany

**Keywords:** federated queries, feasibility study, Fast Healthcare Interoperability Resource, FHIR Search, CQL, ontology, terminology server, query, feasibility, FHIR, terminology, development, COVID-19, automation, user interface, map, input, hospital, data, Germany, accessibility, harmonized

## Abstract

**Background:**

The COVID-19 pandemic highlighted the importance of making research data from all German hospitals available to scientists to respond to current and future pandemics promptly. The heterogeneous data originating from proprietary systems at hospitals' sites must be harmonized and accessible. The German Corona Consensus Dataset (GECCO) specifies how data for COVID-19 patients will be standardized in Fast Healthcare Interoperability Resources (FHIR) profiles across German hospitals. However, given the complexity of the FHIR standard, the data harmonization is not sufficient to make the data accessible. A simplified visual representation is needed to reduce the technical burden, while allowing feasibility queries.

**Objective:**

This study investigates how a search ontology can be automatically generated using FHIR profiles and a terminology server. Furthermore, it describes how this ontology can be used in a user interface (UI) and how a mapping and a terminology tree created together with the ontology can translate user input into FHIR queries.

**Methods:**

We used the FHIR profiles from the GECCO data set combined with a terminology server to generate an ontology and the required mapping files for the translation. We analyzed the profiles and identified search criteria for the visual representation. In this process, we reduced the complex profiles to code value pairs for improved usability. We enriched our ontology with the necessary information to display it in a UI. We also developed an intermediate query language to transform the queries from the UI to federated FHIR requests. Separation of concerns resulted in discrepancies between the criteria used in the intermediate query format and the target query language. Therefore, a mapping was created to reintroduce all information relevant for creating the query in its target language. Further, we generated a tree representation of the ontology hierarchy, which allows resolving child concepts in the process.

**Results:**

In the scope of this project, 82 (99%) of 83 elements defined in the GECCO profile were successfully implemented. We verified our solution based on an independently developed test patient. A discrepancy between the test data and the criteria was found in 6 cases due to different versions used to generate the test data and the UI profiles, the support for specific code systems, and the evaluation of postcoordinated Systematized Nomenclature of Medicine (SNOMED) codes. Our results highlight the need for governance mechanisms for version changes, concept mapping between values from different code systems encoding the same concept, and support for different unit dimensions.

**Conclusions:**

We developed an automatic process to generate ontology and mapping files for FHIR-formatted data. Our tests found that this process works for most of our chosen FHIR profile criteria. The process established here works directly with FHIR profiles and a terminology server, making it extendable to other FHIR profiles and demonstrating that automatic ontology generation on FHIR profiles is feasible.

## Introduction

### Background

Researchers require data to test, refine, and improve their models. Historically in health care, these data have often only been accessible and discoverable locally. Due to different protocols, proprietary solutions, and missing terminology, there is a lack of standardization to promote interoperability and data reuse [1].

In a national effort, the Medical Informatics Initiative (MII) in 2017 started to establish a national research platform for health care professions [2]. Local data integration centers (DICs) collect the vast amount of health care data from the clinics and make them accessible across institutional boundaries. The DICs provide different services, such as data integration, data harmonization, standardized data repositories, consent management, and ID management, and form the backbone of a cross-institutional research network.

Data harmonization is achieved by applying Health Level 7 (HL7) Fast Healthcare Interoperability Resources (FHIR), which is an interoperability standard for health care information [3]. It defines a common health care business entity model with *Resources* as basic building blocks. Each *Resource* has a defined set of data elements, constraints, and relationships to other *Resources*. Common *Resources* relevant to clinical researchers are *Patient*, *Observation*, *Condition*, *Procedure*, *MedicationStatement*, *Consent*, and *Immunization*. FHIR profiles can further constrain and extend the predefined *Resources* for specific use cases.

The COVID-19 pandemic revealed the urgency of addressing the interoperability challenge [4]. The German Corona Consensus Dataset (GECCO) [5] and its representation in FHIR profiles were developed to address the semantic interoperability challenge on a national level.

GECCO consists of 83 data elements defined in FHIR profiles that characterize COVID-19 patients according to their medical history, findings, demographics, laboratory values, medications, symptoms, therapy, and vital signs. Each profile’s *Bindings* to *ValueSets* (defined sets of medical terminology) that reference the *CodeSystems* Systematized Nomenclature of Medicine-Clinical Terms (SNOMED CT), Logical Observation Identifiers Names and Codes (LOINC), *International Classification of Diseases and Related Health Problems, 10th edition*, German version (ICD-10-GM), and Anatomical Therapeutic Chemical (ATC) [5] define the medical terms associated with COVID-19 patients within the German health care system. The data set is under ongoing development.

In the CODEX project funded by the German Federal Ministry of Education and Research (BMBF), the existing infrastructural progress of the MII is the foundation to create a web-based federated query tool, which researchers can use for cohort discovery/feasibility queries based on the GECCO data model.

**Figure 1:**
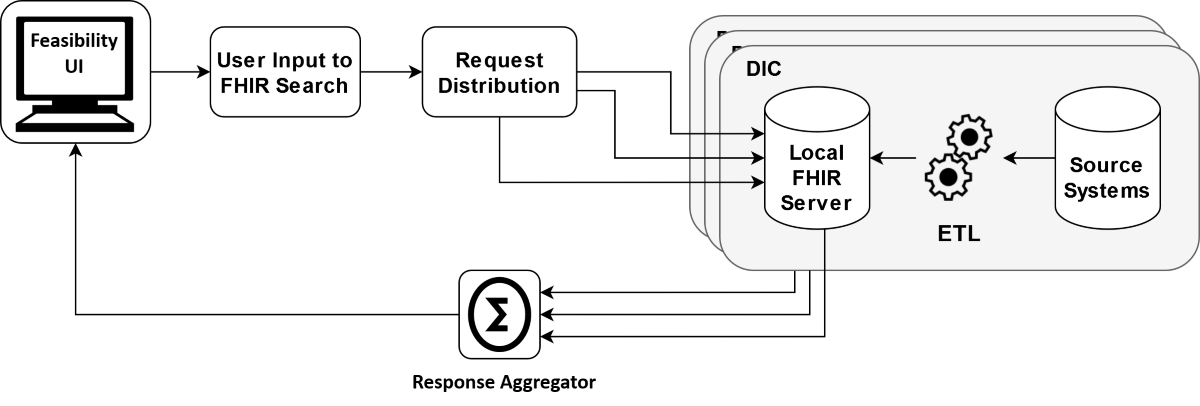
CODEX feasibility architecture. DIC: data integration center; ETL: extract, transform, and load; FHIR: Fast Healthcare Interoperability Resources; UI: user interface.

Within the CODEX feasibility architecture (Figure 1), all German university hospitals extract, transform, and load (ETL) their COVID-19 patient data from their primary source systems to a local FHIR server in GECCO format. Feasibility queries created in the central CODEX feasibility user interface (UI) are forwarded via the CODEX feasibility platform to the decentralized FHIR server within the DICs. Their responses are then transported back to the feasibility UI and displayed to the user, anonymized and aggregated. The detailed architecture is described in a separate publication [6].

The feasibility platform developed within the CODEX project is independent of the COVID-19 use case. Within the FHIR server, arbitrary data can be stored if ETL processes exist to convert the clinical source systems data to FHIR. Furthermore, the query languages (FHIR Search and Clinical Quality Language [CQL]) used at the DICs are universally applicable for arbitrary FHIR data. The highly reusable nature of the infrastructure lends itself well to developing a UI that is use-case-independent. The structure of feasibility queries is consistent—only the use-case-specific query criteria need to be identified. Therefore, for our use case, the data elements within GECCO need to be provided to the user as query criteria.

Extracting criteria from structured data based on a clinical data model for a visual representation on a query interface was also performed by Haarbrandt et al [7] for the openEHR format (where “EHR” refers to “electronic health record”). Contrary to their approach, we keep the FHIR data in their existing format and do not rely on ETL processes. Similar to other federated approaches [8-10], we create feasibility queries centrally and distribute them to the clinical sites. In contrast to them, our feasibility platform is based on FHIR profiles.

### Aim

The complex nature of FHIR profiles makes them unsuitable as a direct interaction format for researchers. This study investigates the use of FHIR profiles, using the GECCO profile as an example, to automatically generate an ontology that provides a generic UI with all the information needed to create feasibility queries and execute them at the hospital sites. We use the term “ontology” following Informatics for Integrating Biology & the Bedside (i2b2; i2b2 Foundation Inc) to refer to hierarchically structured concepts that allow users to create queries using the concepts as criteria [7].

## Methods

### Overview

The aim of generating an ontology is to make criteria findable and identifiable by researchers. These criteria are often independent of how data are stored and processed on a technical level. To bridge this gap, this study investigated not only how to generate an ontology for a UI but also how a mapping and a terminology tree file can be automatically generated to support FHIR request generation.

Thus, we divided the investigation of the problem into 2 parts:

- Creating UI profiles for the visual representation in the UI
- Creating a mapping and a terminology tree for query translation

### UI Profiles

**Figure 2:**
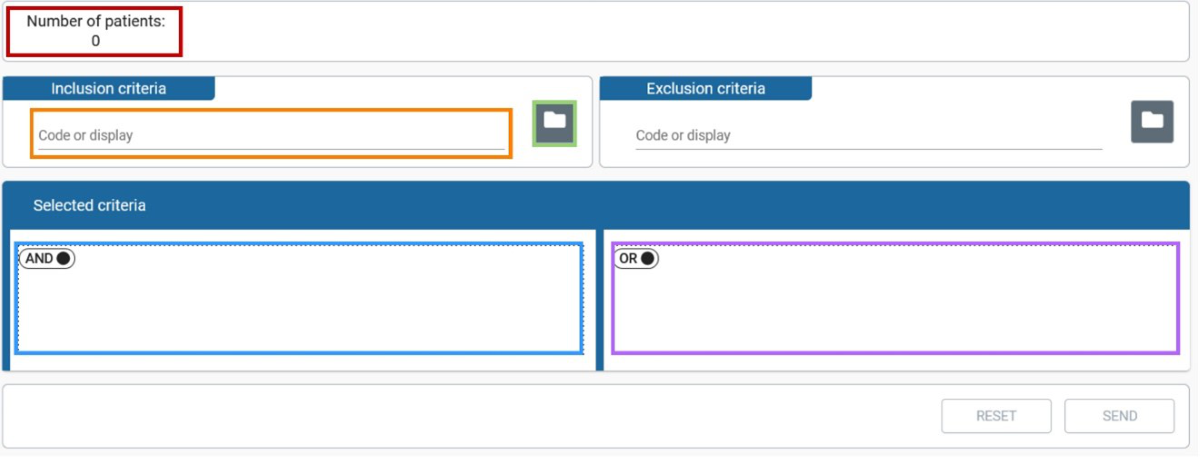
The Codex feasibility UI containing widgets to choose criteria and to create suitable queries. UI: user interface.

The UI (Figure 2), designed for feasibility queries, allows the user to select inclusion and exclusion criteria. The criteria can be chosen from a tree representation (green) or searched for directly (orange). Inclusion and exclusion criteria are presented in a drag-and-drop area where different criteria can be joined using the Boolean AND and OR operations and moved from inclusion to exclusion and vice versa (blue and purple). The represented concepts can be stand-alone or further specified by the user with a value while defining the query. The criteria the user can choose from in the CODEX project are based on GECCO.

The GECCO profiles are defined as FHIR *StructureDefinitions* and can be obtained from Simplifier [11]. Each profile can be regarded as a blueprint of possible *Resource* instance data stored in the DICs.

**Figure 3:**
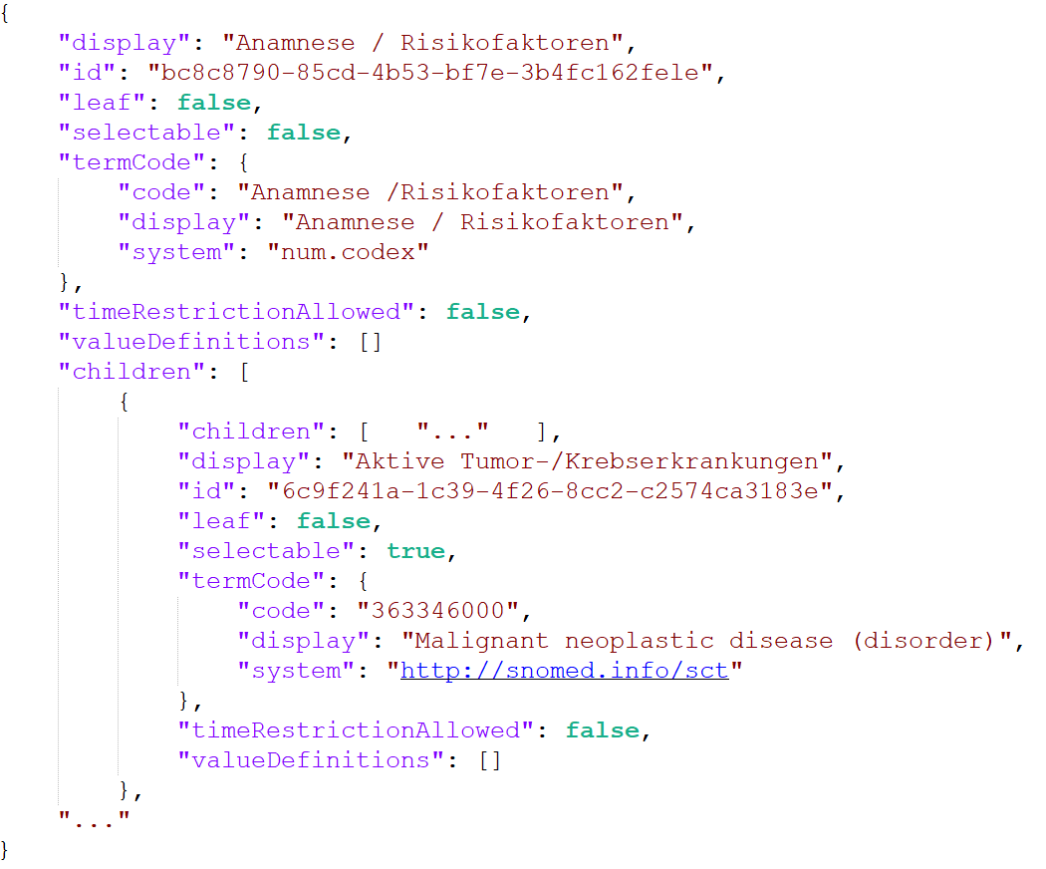
UI profile excerpt. UI: user interface.

A profile analysis must provide uniquely identifiable elements and values of interest that define the criteria to create the ontology for the user. Manual maintenance of such an ontology would be a time-consuming, error-prone, and laborious task [12]. Given the structured nature of the FHIR profiles, an automated approach can be used to generate the ontology. For this purpose, we implemented a Python script [13], which creates a JavaScript Object Notation (JSON) representation of the ontology—the UI profiles (see Figure 3 for an excerpt). This representation puts all criteria in a hierarchical context using a *children* element for each criterion and provides the UI with all the necessary information to display each criterion. If the *children* element is empty, the criterion is a *leaf* criterion, which does not need to be expanded further.

**Figure 4:**
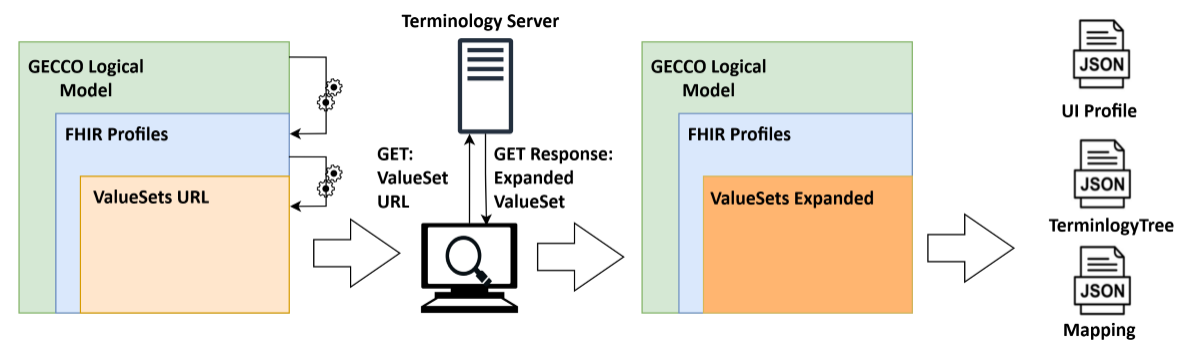
Processing of the GECCO profile to UI profile, mapping, and terminology tree. First, the FHIR profiles are identified within the *LogicalModel*. Next, the *ValueSets* defined in the *Bindings* of specific attributes within the FHIR profiles are identified. Afterward, the *ValueSets* are expanded utilizing a terminology server. Finally, the combined information from the *LogicalModel*, the FHIR profiles, and the expanded *ValueSets* gets processed and converted to the UI profile, the mapping, and the terminology tree. FHIR: Fast Healthcare Interoperability Resources; GECCO: German Corona Consensus Dataset; JSON: JavaScript Object Notation; UI: user interface.

Figure 4 illustrates the entire program's procedure. Besides the UI profile, a mapping and a terminology tree were created.

In addition to the *StructureDefinitions*, the GECCO profile provides a *LogicalModel*. FHIR logical models serve the purpose of collecting requirements from medical experts without having to adhere to the FHIR specifications in the early stages of profile development. In later stages, the elements within the *LogicalModel* can be mapped to the *StructureDefinitions.* For us, the JSON representation of the *LogicalModel* served to identify the categories of our UI. For each category, the *LogicalModel* further defined a set of logical criteria. The name of each criterion was then used to identify the respective profile representing the criteria. Not every GECCO profile needs to be handled individually. The implementation effort can be drastically reduced by grouping all profiles based on the FHIR *ResourceType*.

Each criterion is specified by a code from a terminology system. An optional value allows further restricting the criteria. If no value is provided, the existence of the code is the criterion.

An in-depth analysis of the FHIR profiles allowed us to identify the attributes that specify the criteria and their values. Table 1 displays the attributes of a FHIR profile, which specify the criteria and the values for each FHIR *ResourceType*. In total, 75 (90%) of the 83 defined profiles could be represented in this fashion.

**Table 1:** Identified attributes that specify the concepts and values for the criteria.

*ResourceType*	Criteria-specifying attribute	Value-specifying attribute	Example
*Condition*	code		Type 2 diabetes mellitus
*Observation* (concept)	code	value (*CodeableConcept*)	Sex assigned at birth: female
*Observation* (quantity)	code	value (*Quantity*)	Weight: 70 kg
*Procedure*	code		Plain radiography (procedure)
*MedicationStatement*	code		Product containing antipyretic
*Immunization*	vaccineCode		Typhus vaccine (product)
*DiagnosticReport*	code	conclusion	Diagnostic imaging study: radiological finding characteristic for COVID-19
*Specimen*	type		Blood specimen

Some profiles with the same *ResourceType* do not hold the information on the value in the same attribute. For these cases, additional heuristics or corner cases need to be established. One reoccurring case in this regard is the representation of the *Observation* *Resource*. FHIR does not differ between *Observations* that have recorded a concept or a value. For example, the concept of smoking status is defined as an *Observation* with values indicating the smoking frequency. The body height is also defined as an *Observation* but has a quantity as a value. Therefore, different UI profiles and mappings are needed.

The profile itself is not a criterion. Instead, the profile's criteria-specifying attribute (see Table 1) defines the set of criteria, and the profile specifies how all criteria within this set will be modeled.

After identifying the *ResourceType,* the set of criteria and possible values for each criterion can be resolved using the *Bindings* of each specifying attribute. Each *Binding* contains the canonical URL of a *ValueSet*. A *ValueSet* defines a set of medical terms from medical terminology, such as ICD-10. An instance of the Ontoserver, a terminology server [14] based on the FHIR standard, administers all *ValueSets* from the GECCO profile. After identifying the *ValueSet*, the available values can be obtained from the terminology server using the *expand* operation. Each concept in the *ValueSet* has a unique combination of code and system, which identifies the criterion. The concepts are represented in a list. To build our ontology, we derived the hierarchy of codes based on the is-a relationship between them. We further enriched our ontology with information about how the criteria should be represented (ie, which criterion is selectable).

To illustrate the process, take the criteria group “Chronic Lung Disease” with the parent category “Anamnesia/Risk Factors.” The profiles JSON is analyzed and based on the field *ResourceType*, identified as a *Condition* whose attribute “code” (contains a code and a system) defines the criterion. Other attributes are in this case not of primary interest to the researcher and can be ignored during query processing or set to specific values for the most common research interest, like only searching for verified conditions.

Valid codes can be obtained from SNOMED-CT and ICD-10-GM *ValueSets*. Currently, valid codes are only displayed for codes from a single *CodeSystem* due to the potential confusion caused by the overlap of concepts between *CodeSystem* (ie, sleep apnea is part of ICD-10 and SNOMED CT). The ICD-10-GM *CodeSystem* is chosen because of its broader adaptation in clinics. The *ValueSet* is transformed into a tree structure based on the subsumption relations within the terminology and appended below the “Chronic Lung Disease” node.

### FHIR Search/CQL

Between its visual representation and the execution as a FHIR Search request at the university hospital sites, the feasibility query created in the UI is sent to the backend in an intermediate data format. The intermediate query format was developed within the CODEX project and is named Structured Query (SQ).

Like the UI, the SQ is composed of 2 parts:

- The inclusion criteria are in conjunctive normal form without negation.
- The exclusion criteria are in disjunctive normal form without negation.

They are combined in an AND NOT expression:

SQ=inclusion criteria (CNF)⋀¬ exclusion criteria (DNF)

The use of an intermediate format simplifies the translation into multiple query languages. FHIR *Resources* can be requested using FHIR Search or CQL. FHIR Search uses GET requests to obtain *Resources* from an FHIR server. All *Resources* define a set of search parameters that can be used to filter the search result.

FHIR Search has limitations in its expressiveness. It requires defined search parameters and cannot express inclusion and exclusion criteria in a single query [15].

Although these issues have been overcome within the CODEX project through workarounds including custom search parameters, multiple FHIR Search requests, and combination logic of the results, CQL presents a promising solution to overcome the limitations of FHIR Search [16].

### Mapping

To allow for a high degree of modularity we applied the software design pattern Separation of Concerns [17]. This allowed for independent development of the components and provided more flexibility to adjust to individual sites’ existing infrastructure and future developments. The UI is separated from the query process and the query language, allowing high maintainability. Therefore, the UI profiles do not hold information on the underlying FHIR data model or the query languages. Furthermore, the hierarchic information is not transferred in the SQ, allowing for independent ontology development.

Therefore, the lost information about the *Resources* and their search parameters needed to create the FHIR Search request at the clinical server side must be reintroduced.

**Figure 5:**
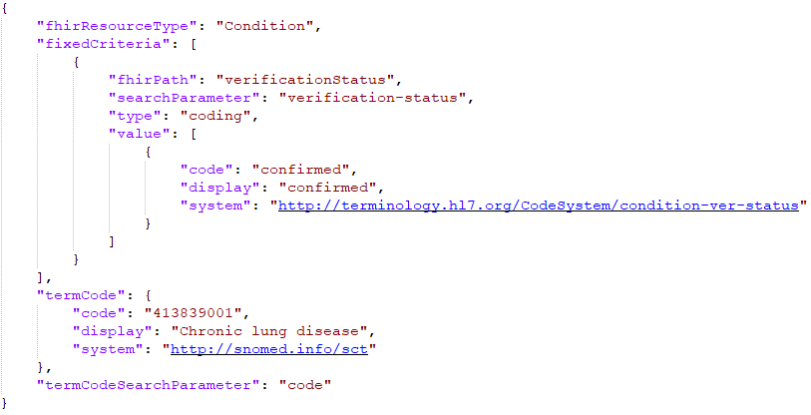
Mapping entry for "Chronic Lung Disease." The search parameter for the code identifying the criterion is "code." The value of *verificationStatus* is fixed to “confirmed.”

To achieve this, we created a mapping for each criterion (Figure 5), storing all information needed to translate the SQ into FHIR Search and CQL requests.

Again, we used the same process as established previously to generate the UI profiles. Instead of rendering the codes for the criteria- and value-specifying attributes, we linked the codes and the search parameter and FHIR paths for the same criteria.

Utilizing the criteria code as a key, we specified the search parameters for the code and the value.

Not all attributes of a FHIR profile have a default search parameter, especially all *Extensions* as they are not part of the official FHIR standard. To handle these cases, additional (custom) search parameters needed to be defined, added to the FHIR server, and referred to in our mapping.

We further defined so-called fixed criteria to restrict attributes not available to the user by setting their search parameter to a predefined value. This is necessary, for example, to only search for confirmed diagnoses.

For the chronic lung disease example, a mapping entry was created for each chronic lung disease code with the corresponding information that the code can be found within the resource *Condition* under the search parameter “code” with a fixed criterion “verification-status” with the value “confirmed” (see Figure 5).

Criteria that are not *leaves* in the ontology tree represent all criteria that descend from it. The subcriteria are not sent in the SQ and need to be resolved at the clinical sites. Due to the lack of terminology servers, we provided the terminology tree JSON file, which represents the UI profiles reduced to only hierarchic information between codes. A terminology tree consists of nodes with 2 properties: the code that identifies the concept within the tree and a list of child nodes.

## Results

### Corner Cases

The established process can parse all profiles defined in GECCO. However, in an in-depth analysis of the GECCO profiles, we identified 7 corner cases needing explicit handling, increasing the implementation effort. Table 2 lists the issues preventing the handling based on *ResourceType*. Explicit handlings were implemented for each case.

Using the explicit and *ResourceType*-based mapping, we successfully created the UI profiles, mapping, and terminology tree for the additional 7 corner cases, thus covering a total of 82 (99%) of 83 profiles. Only the date of birth was excluded due to privacy concerns but could have been implemented in a similar manner.

Examples of the feasibility UI with the loaded UI profiles and an example query can be found in Multimedia Appendix 1.

**Figure 6:**
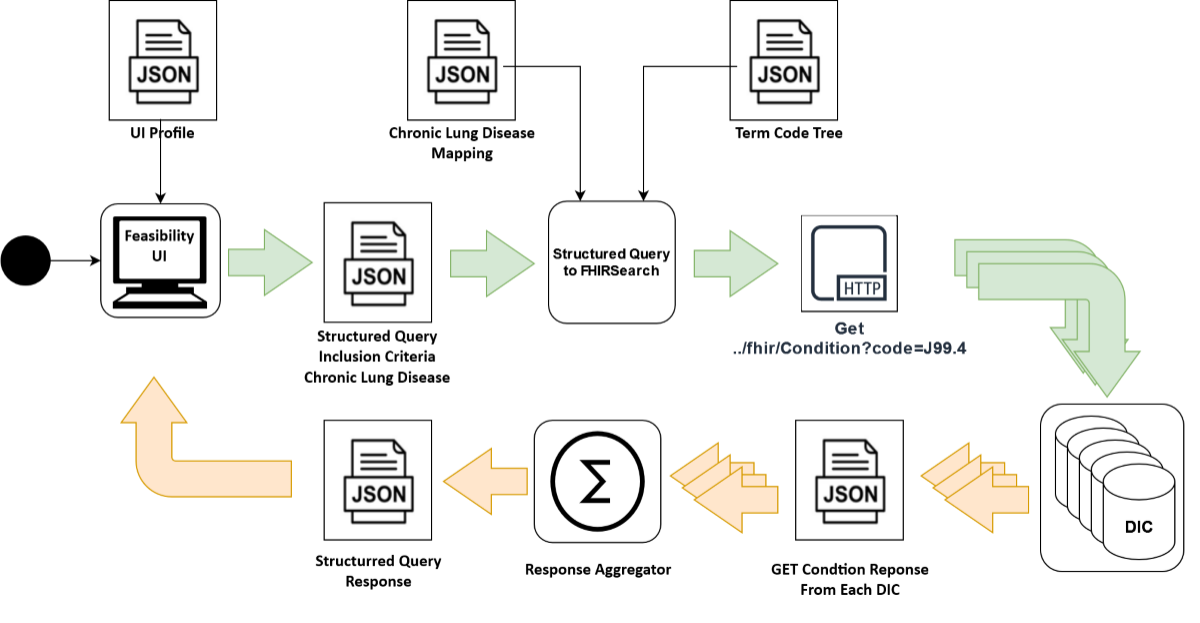
Activity diagram showcasing the creation and execution of the feasibility request based on the UI profiles in the CODEX feasibility architecture. DIC: data integration center; FHIR: Fast Healthcare Interoperability Resources; JSON: JavaScript Object Notation; UI: user interface.

The overall architecture utilized the results as shown in Figure 6.

The criteria were selected and combined into a feasibility query based on the UI profiles. The resulting SQ was sent to a back-end component, which translated the SQ utilizing the mapping. The resulting FHIR Search requests were distributed to all DICs at the clinical sites and executed on the GECCO-harmonized data using the mapping and terminology tree we generated. The responses were aggregated, anonymized, and sent back to the feasibility UI to display the result.

**Table 2:** Corner cases, by their profile name and *ResourceType*, and the issue preventing the default handling.

Profile	*ResourceType*	Issue preventing default handling
Sequential Organ Failure Assessment (SOFA)	*Observation*	The value of the SOFA score is stored in value[integer], not in value[quantity].
History of Travel	*Observation*	The information of interest is stored in a component.
Systolic/Diastolic Blood Pressure	*Observation*	The information of interest is stored in a component. Contrary to the “History of Travel,” “Systolic/Diastolic Blood Pressure” is stored as a quantity, not as a concept.
Covid-19 Symptoms	*Condition*	For COVID-19 symptoms, we decided that the severity should also be settable by the researcher as a value.
Ethnic Group	*Extension*	The ethnic group is an extension and needs a specific search parameter and FHIR^a^ path.
Age	*Extension*	Age is an extension and needs a specific search parameter and FHIR path.

^a^FHIR: Fast Healthcare Interoperability Resources.

### Evaluation

At the time of writing this publication, the DICs were still under development, and the ETL processes to fill the FHIR servers with real-world GECCO data have yet to be rolled out. Many hospital sites use the electronic data capture tool REDCap [18] to collect COVID-19 patient data and the ODM2FHIR tool [19] to transform the data to FHIR. For our automated and manual tests, we used this toolchain to create our test patients. The manual tests were conducted by selecting logical combinations from the criteria defining the test patient in the UI. In addition, we generated SQs that request each criterion and should return our test patient as a result. The test data and the generated SQs are available in Ref. [20].

In 6 (7%) of 84 conducted manual tests, a discrepancy between the test data encoding and the available elements in the UI made it impossible to obtain the data of interest. The 6 discrepancies were caused by 4 different sources of errors:

- SNOMED CT postcoordination: SNOMED CT makes it possible to specify concepts (eg, defining the body side of a finding) using postcoordinated expression (PCE) [21]. PCE-coded concepts represent a subset of a non-PCE-coded concept but are not part of expanded value sets if not explicitly defined. In consequence, only the non-PCE-coded concept is available in the UI.
- GECCO version discrepancies: Although GECCO version 1.0.4 was used as the basis for the UI implementation, the test data is still based on the previous version 1.0.3. This discrepancy sometimes results in different coding for the concepts.
- Unit definitions: The *LogicalModel* of GECCO defines units for all quantitative values. The current implementation does not allow converting between units. Users must search the unit according to the test data, leading to errors in 2 cases where the unit is unavailable.
- *CodeSystem* discrepancies: Although the GECCO profile allows for values from different *CodeSystem*, we reduced this complexity to values from a single *CodeSystem*. Not for every value does a corresponding code in all *CodeSystem* exist. Consequently, some codes in the test data are not available in the UI.

## Discussion

### Principal Findings

We presented the automatic generation of an ontology for a federated feasibility search tool and the necessary information to translate an intermediate query format to FHIR Search and CQL. We based the generation of the ontology, and the mapping, on FHIR profiles, allowing us to generalize our method to FHIR profiles, which represent a concept with a unique identifying code and an optional value. We successfully implemented UI profiles (UI representations) as well as the mapping for all criteria from GECCO and verified our solution based on an independently developed test patient.

We use FHIR data in their original format while simultaneously representing the concepts as criteria in a simplified model for the end user, resulting in a reduced technical burden, which improves usability. Other ETL processes on the FHIR data are unnecessary. Further, we generated the ontology automatically and did not rely on manual maintenance. Consequently, the development time of an ontology can be drastically reduced, and the ontology can be adapted rapidly to version changes of the data set.

### Related Work

The development of a feasibility portal for medical health data poses an ill-structured problem. A wide opportunity space holds solutions in different architectures, data formats, query languages, and tooling.

A federated approach is the greatest common feature between existing feasibility solutions to overcome legal boundaries and ensure privacy protection on sensitive health care data. For proprietary, i2b2, and Observational Medical Outcomes Partnership (OMOP) data, solutions exist that provide researchers with an ontology-based UI [9,10,22]. These platforms can also be utilized for FHIR and openEHR data but require additional ETL processes [7,23]. The Leaf project [8] presents an alternative approach by using a model agnostic query system for medical data stored in Structured Query Language (SQL) databases. Like our approach, an ontology holds the information on the criteria available to the user, and similar criteria are mapped to WHERE clauses for SQL statements. To apply their query system to FHIR requires a flat representation of the FHIR *Resources* in a SQL database. As the used FHIR servers at the DICs do not store flattened representations of the FHIR profiles and an additional representation in flattened form would cause data redundance, their solution could not be applied to our problem. Regardless, an ontology and a mapping would have also been needed to utilize the Leaf approach. Other existing solutions utilizing the FHIR standard for federated feasibility queries rely on computer scientists to transfer their research questions to FHIR Search, CQL, or SQL [24,25]. Existing FHIR-based federated feasibility query tools with a graphical UI, developed for health care professionals, rely on manual curation of search criteria [26,27]. Manual curation is a laborious task and can take years.

With the presented work, we provide a solution for creating an ontology based on FHIR profiles suitable for medical professionals to create and execute federated feasibility queries for data in FHIR format.

### Lessons Learned

The presented methodology relies on the extensive investigation of the FHIR profiles. Often, the expertise in those lies with the domain experts and modelers. Software developers must not only identify handling for individual *Resources* based on FHIR types but also discover all corner cases. A more interdisciplinary team could facilitate and shorten the development process. The presented implementation for GECCO can act as a starting point for other FHIR profiles. Developers need to add handling for *ResourceTypes* that are not yet implemented and add corner cases for profiles that do not align with the default handling.

The development and especially the delivery of the ontology rely on the infrastructure at the clinical sites. The Blaze FHIR server [28] implementation utilized in this project allowed the usage of CQL and custom search parameters. In contrast, a lack of terminology servers at the sites resulted in the need to make the ontology available in a proprietary format and prevented using the *below* modifier a terminology server offers. In the future, the definition of custom search parameters should be part of the profiling process to ensure that the criteria defined in GECCO are queryable.

### Limitations

Further improvements can be made to our solution to address the issues found. The SNOMED CT postcoordination limitations can be addressed by using the *below* modifier in FHIR Search requests. The *below* modifier resolves the is-relation between the PCE and the non-PCE equivalent but requires a SNOMED CT *CodeSystem* at every site.

Given the ongoing development and fixes in GECCO, our static approach for the UI profiles currently limits the use to a single version. Given the federated nature of the project, we cannot guarantee that every site uses the newest version. Therefore, support of multiple versions would be helpful. Improvements can be made by utilizing the terminology server in conjunction with versioning at run time to create the UI profiles semidynamically.

For usability, the units provided should be converted to the units used at each site during query execution. Research efforts to address this issue can be found in Ref. [29].

The flexible use of values from different *CodeSystems* represents the most significant challenge, as it cannot be solved on a purely technical level. Reducing the values provided to values from a single *CodeSystem* serves to simplify the presentation for the user. Concepts repeated in different *CodeSystems* are listed only once in the UI (eg, sleep apnea is available in ICD-10-GM and SNOMED CT but can only be selected as an ICD-10-GM concept). A mapping between all codes would be necessary to support both code systems. This mapping requires medical expertise as not all concepts can be as directly matched as the example. Stricter profiling with values limited to a single *CodeSystem* would have resulted in a higher workload at each site but improved organizational interoperability. Narrowing the optionality reduces the complexity, ultimately leading to better interoperability [30].

### Future Directions

The high adaptability of the developed platform and the presented methodology open possibilities for a wide range of future work. Applying the presented approach to other FHIR data sets is part of ongoing work in the successor project of CODEX, ABIDE [31], where the same approach is applied to the MII core data set [32]. For cancer research, the presented approach could also be applied to the data model in Ref. [33].

Regarding FHIR, we want to expand the code value representation by establishing attribute filters that further refine the criteria using multiple FHIR *Resource* attributes.

Beyond FHIR, it would also be of interest to test the adaptability of our approach to other structured health care data. Primarily dependent on the mapping capabilities, we see the potential to use the SQ as an intermediate query language for FHIR and other query languages (ie, Archetype Query Language [AQL]) [34]. Previous research work [35] indicates the feasibility of this idea.

The current representation of the ontology is a proprietary format developed within this project. For better exchange, it should be investigated whether the features of a terminology server can be used to exchange the developed ontology in the standardized FHIR format (ie, using a structure map for the mapping) and dynamically load it from there.

Finally, a mapping between complex FHIR *Resources* and simplified interface patterns should be further investigated. The Release 5 draft of the FHIR standard introduces interface patterns, which could abstract a simplified representation from the FHIR *Resource*. Combined with the FHIR mapping language, a simpler resource data model for querying could be developed by domain experts rather than software developers.

### Conclusion

We demonstrated an automated process to generate an ontology for feasibility criteria based on GECCO profiles, showcasing the feasibility of our approach for FHIR-profiled data. We described how to obtain user-relevant data from the FHIR profiles and how to use the same information to create a mapping to translate an intermediate query language to CQL and FHIR Search.

The underlying platform has been deployed across 33 university hospitals in Germany. Test data were used to evaluate our approach and demonstrate its validity.

We see great generalization potential not only for other FHIR profiles but also for structured health care data in general.

## Appendix

Multimedia Appendix 1 Example of loaded UI profiles in the generic feasibility UI and an example query. UI: user interface.

## Abbreviations

CQL: Clinical Quality Language
DIC: Data Integration Centers
EHR: electronic health record
ETL: extract, transform, and load
FHIR: Fast Healthcare Interrogability Resources
GECCO: German Corona Consensus Dataset
ICD-10-GM: International Classification of Diseases and Related Health Problems, 10th edition, German version
JSON: JavaScript Object Notation
MII: Medical Informatics Initiative
PCE: postcoordinated expression
SNOMED CT: Systematized Nomenclature of Medicine-Clinical Terms
SQ: Structured Query
SQL: Structured Query Language
UI: user interface
