# Supplementary material for: Generation of a Fast Healthcare Interoperability Resources (FHIR)-based Ontology for Federated Feasibility Queries in the Context of COVID-19: Feasibility Study
Source: JMIR Med Inform. 2022 Apr 27;10(4):e35789. doi: 10.2196/35789 (PMC9049646; doi:10.2196/35789)

UI-Profile loaded in the generic user interface

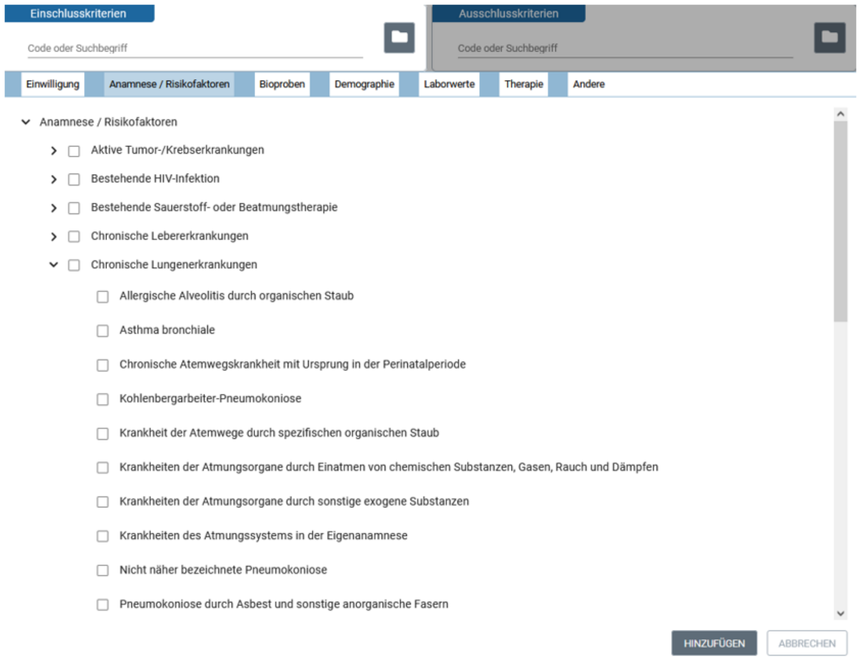


```
"display": "Anamnese / Risikofaktoren",
"id": "bc8c8790-85cd-4b53-bf7e-3b4fc162fe1e",
"leaf": false,
"selectable": false,
"termCode": {
    "code": "Anamnese / Risikofaktoren",
    "display": "Anamnese / Risikofaktoren",
    "system": "num.codex"
},
"timeRestrictionAllowed": false,
"valueDefinitions": []
"children": [
    {
        "children": [    "..."     ],
        "display": "Aktive Tumor-/Krebserkrankungen",
        "id": "6c9f241a-1c39-4f26-8cc2-c2574ca3183e",
        "leaf": false,
        "selectable": true,
        "termCode": {
            "code": "363346000",
            "display": "Malignant neoplastic disease (disorder)",
            "system": "http://snomed.info/sct"
        },
        "timeRestrictionAllowed": false,
        "valueDefinitions": []
    },
"..."
```

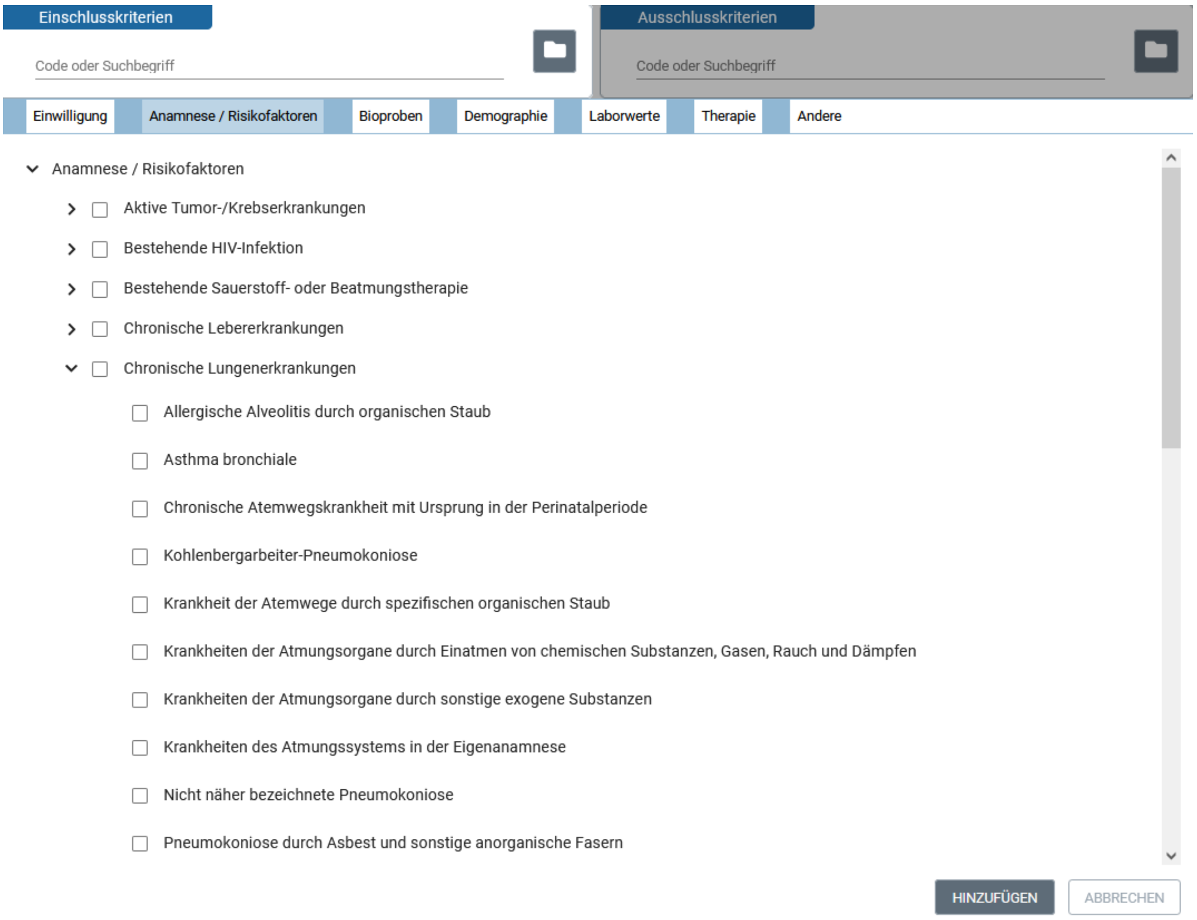

Example of a query created from the ontology

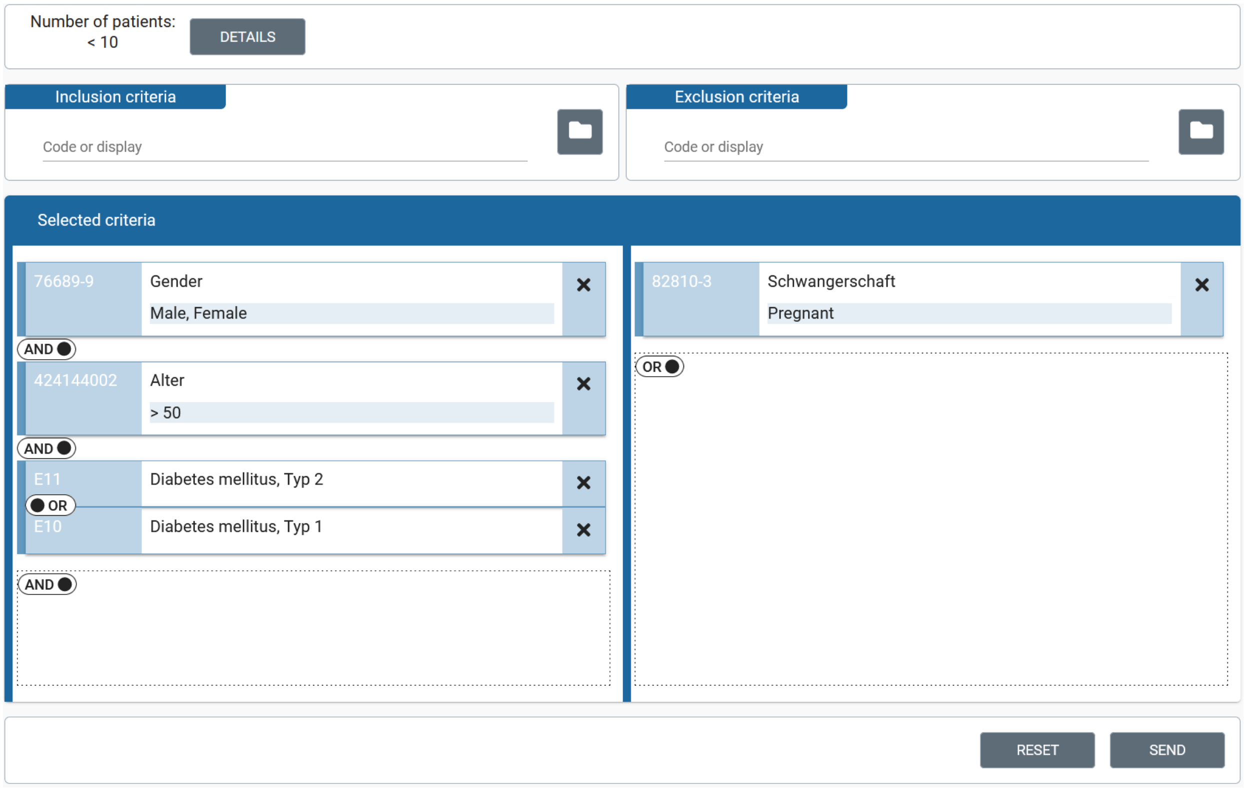

Supplement: Multimedia Appendix 1 [file medinform_v10i4e35789_app1.pdf]
